# *In situ* Metabolic Profiling of Ovarian Cancer Tumor Xenografts: A Digital Pathology Approach

**DOI:** 10.3389/fonc.2020.01277

**Published:** 2020-08-19

**Authors:** Ilaria Piga, Martina Verza, Francesca Montenegro, Giorgia Nardo, Elisabetta Zulato, Tiziana Zanin, Paola Del Bianco, Giovanni Esposito, Stefano Indraccolo

**Affiliations:** ^1^Immunology and Molecular Oncology Unit, Istituto Oncologico Veneto, IOV—IRCCS, Padua, Italy; ^2^Department of Surgery, Oncology and Gastroenterology, University of Padua, Padua, Italy; ^3^Pathology Unit, Istituto Oncologico Veneto, IOV—IRCCS, Padua, Italy; ^4^Clinical Research Unit, Istituto Oncologico Veneto, IOV—IRCCS, Padua, Italy

**Keywords:** ovarian cancer, metabolism, IHC, digital pathology, MCT4

## Abstract

Metabolic profiling of cancer is a rising interest in the field of biomarker development. One bottleneck of its clinical exploitation, however, is the lack of simple and quantitative techniques that enable to capture the key metabolic traits of tumor from archival samples. In fact, liquid chromatography associated with mass spectrometry is the gold-standard technique for the study of tumor metabolism because it has high levels of accuracy and precision. However, it requires freshly frozen samples, which are difficult to collect in large multi-centric clinical studies. For this reason, we propose here to investigate a set of established metabolism-associated protein markers by exploiting immunohistochemistry coupled with digital pathology. As case study, we quantified expression of MCT1, MCT4, GLS, PHGDH, FAS, and ACC in 17 patient-derived ovarian cancer xenografts and correlated it with survival. Among these markers, the glycolysis-associated marker MCT4 was negatively associated with survival of mice. The algorithm enabling a quantitative analysis of these metabolism-associated markers is an innovative research tool that can be exported to large sets of clinical samples and can remove the variability of individual interpretation of immunohistochemistry results.

## Introduction

Metabolic alterations are recognized hallmarks of cancer (1) and have been described in thousands of publications. Key metabolic alterations described in tumors involve glycolysis, glutamine and lipid metabolism, and they contribute to generate ATP that is required for cell proliferation and simultaneously represents a source for macromolecule synthesis and for the replenishment of reactive oxygen species scavenging systems (2). From a general perspective in the context of solid tumors, we can distinguish metabolic alterations of cancer cells from those of the stroma, including endothelial cells (3), fibroblasts (4), and adipocytes (5), as well as those of mobile cells, such as lymphocytes, macrophages, and specialized subpopulations of myeloid cells (6). These two components interact with each other and with the extracellular matrix, and these interactions can take the form of either metabolic competition or metabolic symbiosis. An additional feature of tumor metabolism is represented by its heterogeneity, which can be accounted for by (1) cancer cell autonomous factors, (2) local microenvironment factors such as hypoxia and acidosis, and (3) external factors, including diet, the microbiome, and certain drugs that can generate signals which modulate metabolism in the tumor microenvironment (7).

Advanced technologies, including metabolomics (8) and metabolic flux analysis (9), are key to decode the heterogeneous metabolic preferences and dependencies of tumors *in vivo*, but they can only be performed in a very limited number of patients given the high costs of the equipment and the level of specialization of personnel involved in this type of analysis. Moreover, these techniques do not enable to study intra-tumor metabolic heterogeneity as they assess levels of metabolites in whole tumor lysates or track the incorporation of a labeled substrate into downstream metabolites.

Parallel to these high-tech approaches, which remain fundamental for basic research studies, it is important to evaluate *in situ* biomarkers of dysregulated cancer metabolic pathways which could be analyzed in standard laboratories on archival samples. One possibility is represented by certain transporters or enzymes which, according to many studies, are key for the activity of the underlying metabolic pathway, such as monocarboxylate transporter 4 (MCT4) for glycolysis (10, 11), glutaminase (GLS) for glutamine metabolism (12), and a few others. The protein expression levels of these markers can be easily assessed by immunohistochemistry (IHC), the signal being digitalized and quantified by digital pathology techniques at a reasonable cost per sample (13). The integration of signal quantification into appropriate mathematical models can then be used to define cutoff values in order to stratify samples into biomarker positive or negative and eventually investigate their prognostic or predictive value.

In this study, we tested this hypothesis by staining, for a panel of representative metabolism-associated markers, a set of patient-derived ovarian cancer xenografts (PDXs) and correlated the quantitative expression of these markers with the survival of mice bearing these tumors. Ovarian cancer has a dismal prognosis in most patients because it is often diagnosed at a late stage and cancer cells often become resistant to platinum-based chemotherapy (14). The metabolic traits of ovarian cancer have been reported in several studies which, however, focused on one single aspect of metabolism (15–17). Moreover, there are studies showing that resistance to chemotherapy can be accounted for by certain metabolic features of ovarian cancer cells (18, 19). Stimulated by these considerations, we present here an *in situ* metabolic profiling of ovarian cancer xenografts. The results obtained in this pilot study are hypothesis generating and will be further investigated in patients' samples.

## Materials and Methods

### Patient Data

The studies involving human participants were reviewed and approved by the IOV Institutional Review Board and Ethics Committee (EM 23/2017) and were performed in accordance with the Declaration of Helsinki. The patients/participants provided their written informed consent to participate in this study. Patient-derived xenografts were derived from cancer cells contained in ascitic effusions and obtained from patients bearing epithelial ovarian cancer (EOC). The clinical samples were obtained from either newly diagnosed patients or relapsing patients with EOC at different stages and grades (Table 1).

**Table 1:** Clinical features of the patient-derived xenograft (PDX) utilized in this study.

**Sample ID**	**Histotype**	**Stage**	**Grade**	**Diagnosis/relapse**
PDOVCA 14	Endometrioid	3C	G1	Diagnosis
PDOVCA 15	Serous-papillary	4	G3	Relapse
PDOVCA 17	Serous-papillary	3C	G3	Diagnosis
PDOVCA 24	Serous-papillary	3A	G3	Relapse
PDOVCA 36	Serous	4	G3	Relapse
PDOVCA 39	Serous-papillary	3B	G2	Relapse
PDOVCA 44	Serous-papillary	3C	G3	Diagnosis
PDOVCA 49	Serous-papillary	3A	G1	Relapse
PDOVCA 52	Serous-papillary	3C	G3	Diagnosis
PDOVCA 53	Endometrioid	3C	G3	Relapse
PDOVCA 54	Serous-papillary	4	G1	Relapse
PDOVCA 57	Serous-papillary	3C	G3	Relapse
PDOVCA 58	Serous-papillary	3A	G3	Relapse
PDOVCA 62	Serous-papillary	3C	G3	Relapse
PDOVCA 69	Serous-papillary	3C	G3	Relapse
PDOVCA 70	Serous-papillary	3C	G3	Relapse
PDOVCA 82	Serous-papillary	3C	G3	Relapse

*Histotype, stage, and grade refer to the patient samples from whom PDXs were derived. Diagnosis/relapse indicates that PDXs were established from ascitic fluid samples obtained at diagnosis or relapse, respectively*.

### Generation of Ovarian Xenografts

Tumor cells from the ascitic fluid were isolated as previously described (20). PDXs were obtained and propagated by injecting 1 × 10^6^ tumor cells intraperitoneally into 8-week-old female NOD/SCID mice purchased from Charles River Laboratories (Wilmington, MA, USA) and housed in our specific pathogen-free animal facility. The animals developed solid tumors with a substantial ascitic component at different time points, depending on tumor engraftment and growth. At sacrifice, tumors were harvested by dissection, fixed in formalin, and embedded in paraffin for histology and immunohistochemistry analyses. All procedures involving animals and their care conformed to institutional guidelines that comply with national and international laws and policies (EEC Council Directive 86/609, OJ L 358, 12 December 1987). The animal study was reviewed and approved by the Italian Ministry of Health (n. 217/2013-B).

### Histology and Immunohistochemistry

Three-micron-thick formalin-fixed, paraffin-embedded tumor samples were either stained with hematoxylin and eosin or processed for IHC. In this case, IHC was performed by using an automatic stainer BOND III (Leica Microsystems, Wetzlar, Germany) and by using the following antibodies according to the manufacturer's instructions: anti-ACC rabbit mAb (clone C83B10, dilution 1:100), anti-FAS rabbit mAb (clone C20G5, dilution 1:100), anti-phospho-histone H3 (pHH3) rabbit polyclonal Ab (dilution 1:100), all from Cell Signaling Technology Danvers, Massachusetts, USA; anti-GLS rabbit mAb (clone EP7212, dilution 1:200), anti-PHGDH mouse mAb (clone Ab57030, dilution 1:100) both from Abcam, Cambridge, UK, anti-MCT1 rabbit polyclonal Ab (dilution 1:50; Millipore, Burlington, MA, USA), anti-MCT4 rabbit polyclonal Ab (dilution 1:300; Santa Cruz Biotechnology, Dallas, TX, USA), and anti-mouse CD31 rat mAb (clone SZ31, dilution 1:20; DIANOVA GmbH-Hamburg, Germany).

### Image Acquisition and Analysis

Tumor representation and quality of staining were initially evaluated by one experienced pathologist (GE). The slides were digitally acquired at × 20 magnification by the Aperio CS2 (Leica Biosystems, Wetzlar, Germany), and the evaluation of the IHC score was assessed through the Scanscope Image Analysis software (ImageScope v12.4.0.708). On the basis on their localization, the different markers were analyzed by using the Aperio membrane algorithm v9 (MCT1, MCT4), the Aperio cytoplasmic algorithm v2 (GLS, FAS, PHGDH, and ACC), the Aperio nuclear algorithm (pHH3), and the microvessel analysis v1 (CD31). The Aperio Genie Classifier was trained to recognize tumor tissue, stroma, and background (glass) and then combined with Aperio Membrane v9 and Aperio Cytoplasmic v9. The results provided the percentage of cells with different expressions of proteins classified as 3+ (highly positive), 2+ (intermediate positive), 1+ (low positive), and 0 (negative). In the case of GLS in view of the granular pattern of cytoplasmic staining obtained a two-tier classification system was used: 0 (negative) and 1 (positive). The sum of the percentage of marker-positive cells for these tiers equals 100%. The digital quantification performed by the software was confirmed by the pathologist.

### Immunoblotting Assay

Whole-cell lysates (1 × 10^6^ cells) were prepared in RIPA lysis buffer (Cell Signaling Technology) containing a protease and a phosphatase inhibitor cocktail (Sigma Aldrich, St. Louis, MO, USA). Proteins were quantified using Quantum Protein Assay (EuroClone, Milan, Italy), and about 30 μg were denatured and loaded in a midi polyacrylamide gel 4–12% (Life Technologies). Separated proteins were transferred for 2.5 h at 400 mA on a nitrocellulose membrane (GE Health Care, Glattbrugg, Switzerland). Membranes were saturated overnight at 4°C with Tris-buffered saline−0.1% Tween−5% milk and then incubated with primary antibody, according to the manufacturer's instructions. Immunoprobing was performed using the same antibody described for the IHC assay, and it was followed by hybridization with a horseradish peroxidase-conjugated anti-rabbit or anti-mouse Ab (Perkin Elmer, Waltham, MA, USA). The antigens were identified by luminescent visualization using Western Lightning Plus ECL reagents (Perkin Elmer, Waltham, MA, USA), and signal intensity was detected using UVITEC Alliance Software (Cambridge, UK). Protein expression was assessed and normalized to actin (Sigma Aldrich) as the housekeeping gene.

### Statistical Analysis

Data were analyzed with RStudio (RStudio: Integrated Development for R. RStudio Inc., Boston, MA, US). The quantitative variables were summarized as median and interquartile range. A descriptive analysis of the strength of the relationship between the levels of all the considered markers was performed using Spearman's rank correlation coefficient. The survival times were estimated with the Kaplan–Meier method and compared among groups of markers with the log-rank test. The *P*-values were adjusted for multiple comparisons using the Benjamini–Hochberg method.

## Results

### Selection of Metabolism-Associated Markers and Panel Setup

**Figure 1:**
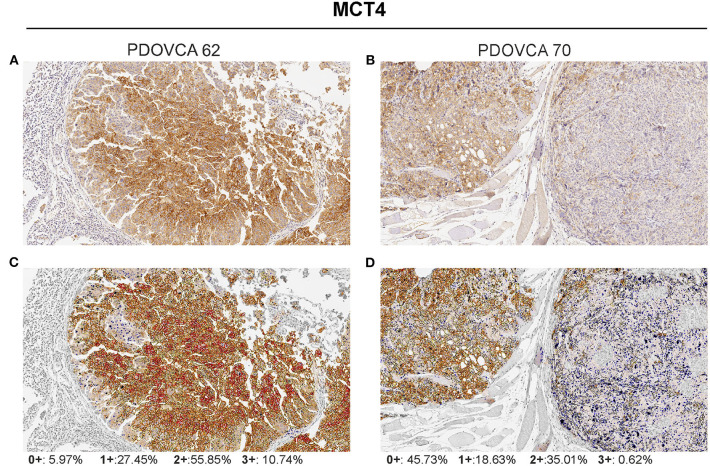
Visualization of the Aperio membrane algorithm to quantify MCT4 expression in tumor. **(A,B)** Two representative samples of patient-derived xenograft with different levels of MCT4 expression (hematoxylin counterstain; original magnification ×20). **(C,D)** The same samples showing final a mark-up of the analyzed tissue. The algorithm utilized automatically detects and classifies cell membrane positivity as strong (3+/red), moderate (2+/orange), or weak (1+/yellow). Negative/0+ cells show only nuclear staining (blue). The gray areas were excluded from the analysis.

**Figure 2:**
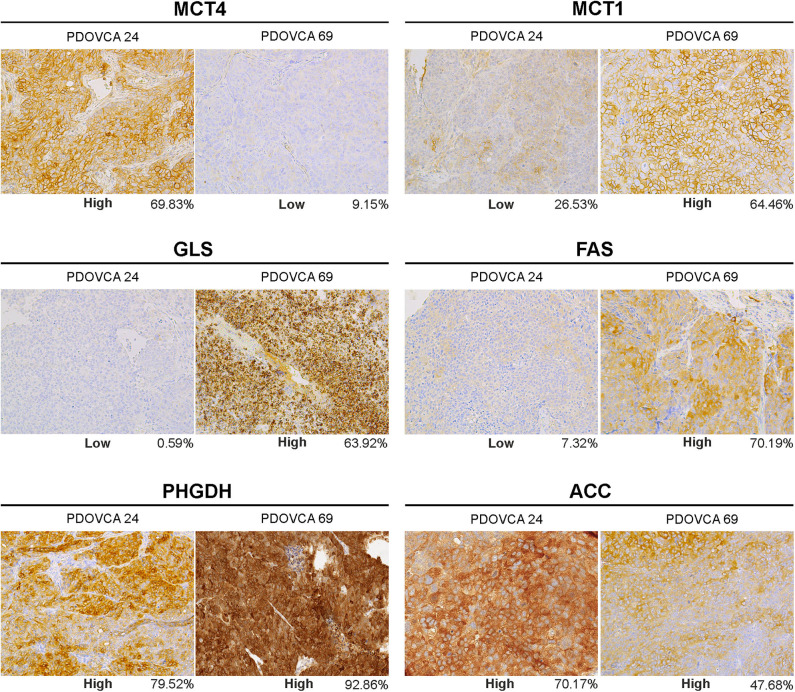
Representative pictures of two patient-derived xenograft samples (PDOVCA 24 and PDOVCA 69) stained for the various metabolism-associated markers: MCT4, MCT1, GLS, FAS, PHGDH, and ACC (original magnification, ×20). The percentage below each panel indicates the sum of 2+ and 3+ values of the marker according to digital pathology analysis.

We selected the following markers to be included in our IHC panel: MCT1, MCT4, GLS, phosphoglycerate dehydrogenase (PHGDH), FAS, and ACC. These markers identify key transporters or enzymes involved in glycolysis (MCT1, MCT4) and in glutamine (GLS), glycine (PHGDH), and fatty acid metabolism (FAS and ACC). For all markers analyzed but GLS, the algorithm first identifies tumor cells and then quantifies the expression levels according to a four-tier classification system (0, 1+, 2+, and 3+) as described in the “MATERIALS AND METHODS” section. In the case of GLS, a two-tier classification system is used. Representative pictures showing the IHC score of two PDX samples stained with anti-MCT4 antibody are shown in Figure 1. For statistical analysis, for each sample we grouped the percentage of cells with 0/1+ and 2+/3+ values. The median value of the percentage of positive cells of all analyzed PDX samples was used to stratify them into two groups based on the expression of a given marker: samples whose quantitative value were above the median value were classified as “high” and those below as “low.” The detailed results of marker expression in the 17 PDXs analyzed are presented in Supplementary Tables 1, 2. Representative pictures of PDX samples stained for the six metabolism-associated markers are shown in Figure 2.

### Association Between Markers

As the markers selected identify key metabolic processes, profiling PDX samples enabled us to investigate possible associations between the markers. This analysis disclosed that MCT4 was negatively associated with FAS (*r* = −0.55). In contrast, MCT1 and GLS1 were positively associated (*r* = 0.41), as well as FAS and GLS1 (*r* = 0.31) and FAS and ACC (*r* = 0.32). No other association was found between the other markers analyzed.

### Association With Survival

**Figure 3:**
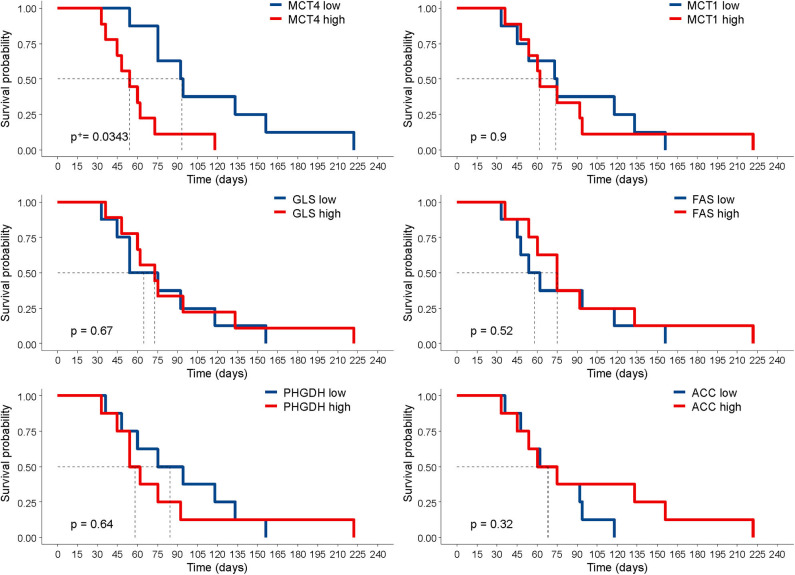
Correlation of marker expression with survival. Kaplan–Meier curves according to the levels of marker expression, dichotomized according to their median value. Statistical significance was calculated with the log-rank test. ^+^*P*-value was adjusted for multiple comparisons using the Benjamini–Hochberg method.

Next, we investigated whether the expression of any of these markers was associated with survival in tumor-bearing mice. The survival of mice is defined by an ethical end-point, i.e., the time when mice have to be euthanized because they develop ascites or show signs of sufferance. In our set of samples, this parameter ranged from 33 to 222 days, depending on the PDX. No anti-tumor drug was administered to the mice in these experiments, and survival time was calculated by averaging the survival of *n* = 3 mice per PDX. The results show that only high levels of MCT4 expression were associated with worse survival in this cohort. None of the other markers analyzed correlated with survival (Figure 3). We asked whether the reduced survival of mice bearing MCT4-positive tumors could be due to increased proliferation, as it is known that a link exists between this metabolic process and proliferation (21). We assessed mitotic cells and found that the expression of the pHH3 marker had a positive association with MCT4 (*r* = 0.72). Since lactate, which is exported by MCT4 from tumor cells, can modulate tumor angiogenesis (22), we stained PDX sections with the endothelial cell marker CD31 and calculated MVD. The results show a positive association between MCT4 and MVD (*r* = 0.56).

### Validation of IHC Results

**Figure 4:**
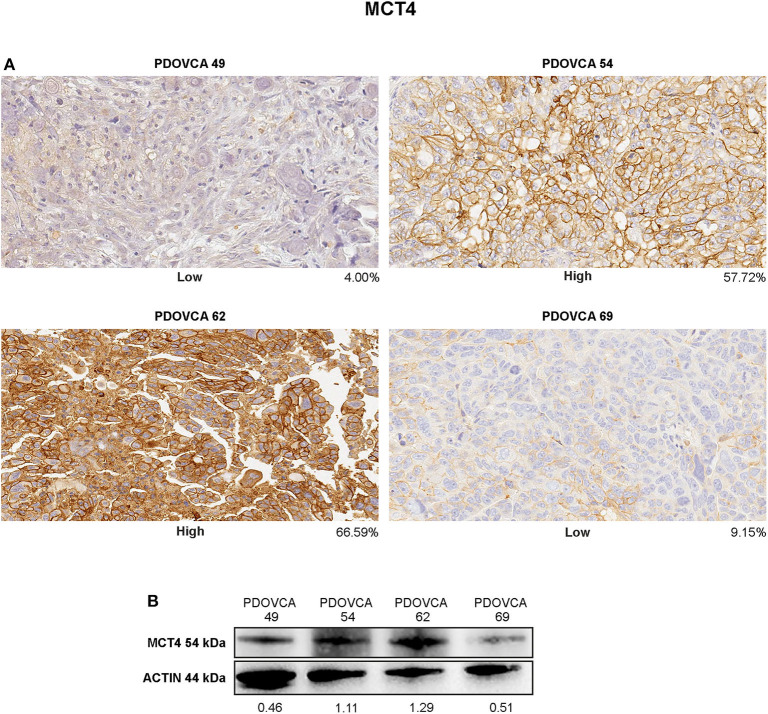
**(A)** Immunohistochemistry staining of MCT4 in representative patient-derived xenograft (PDX) samples showing different protein intensities as assessed by image analysis software on digitalized slides (original magnification, ×20). **(B)** Cell lysates of the same PDX were analyzed in western blot for MCT4 marker. Protein quantification was determined by densitometric analysis, and the values reported in the figure were normalized to actin as loading control.

Finally, we sought to validate the IHC results by an orthogonal technique. To this end, we generated lysates from PDX cells freshly obtained from mice and performed Western blot analysis for the expression of MCT4, the only marker associated with survival in this study. In these experiments, we focused on four PDX samples bearing a high or a low expression of the marker considered, based on quantitative IHC analysis. In the case of the remaining PDXs, Western blot analysis could not be performed due to the lack of tumor lysates available for this assay. Albeit limited by the small number of PDXs analyzed, the results confirmed that the expression levels of the target protein assessed by the IHC-based marker quantification system substantially correlated with those detected in the corresponding tumor lysates by Western blot analysis (Figure 4).

## Discussion

Cancer cells often have increased glycolytic activity compared with normal tissues. In fact, normal cells convert glucose to pyruvate that enters the TCA cycle, whereas cancer cells reduce pyruvate to lactate in order to recycle NADH back to NAD+ to maintain the metabolic flux via glycolysis even in the presence of sufficient levels of oxygen, the so-called Warburg effect (23). This metabolic aberration is considered as a metabolic hallmark of many malignant tumors and, although energetically unfavorable, supports anabolic growth during nutrient limitation (24). This excess of lactate must be expelled from the tumor to the microenvironment in order to prevent cell death via intracellular acidosis. The principal transporter involved in this lactate efflux is MCT4, a member of the H+/monocarboxylate transporter family found to be overexpressed in many types of human cancers, including ovarian cancer (15). Notably, elevated MCT4 expression is associated with decreased overall survival in many cancer types (25).

It is interesting to note that, among the six metabolism-associated markers analyzed, only MCT4 expression was negatively associated with the survival of mice. Highly glycolytic tumors grew faster than poorly glycolytic tumors in these intraperitoneal PDX models, a result which is in line with our previous observations with subcutaneous xenografts of ovarian cancer cell lines (26). MCT4 expression in tumor sections also matched the MCT4 expression levels in cell lysates from PDX cells, confirming the specificity of the antibody used. Previous studies by our group also demonstrated the high correlation between MCT4 expression as evaluated by using this antibody and the glycolytic phenotype of tumor cells both *in vitro* and in mouse models (10). Cancer cells with high MCT4 expression proliferated faster than MCT4-negative cancer cells, according to the results of pHH3 expression in tumor sections. Moreover, the accelerated growth of high MCT4 PDX could also be accounted for by the effects of lactate on the tumor microenvironment, including the promotion of angiogenesis (27), as supported by the strong association between MVD and MCT4 markers. Understanding metabolic reprogramming of tumor cells is fundamental for understanding tumor drug resistance and developing anticancer therapy. We recently reported that glucose-addicted ovarian cancer samples yield better response to platinum-based chemotherapy compared with non-glucose-addicted tumors (19), thus marking the possible clinical implications of the metabolic traits of tumors on drug response.

Cancer cells do not only present alterations in glycolytic phenotype but also of other metabolic pathways interrogated by our panel, including lipid and amino acid metabolism. FAS is the enzyme accounting for the *de novo* synthesis of fatty acids, and it is highly expressed in many human cancers, including ovarian cancer (28). A high expression of FAS provides proliferative and metastatic potential; moreover, the high expression of FAS in EOC is associated with poor prognosis (29).

Another important metabolic pathway altered in cancer cells is glutamine. By using isotype tracer and bio-energetic analysis, Yang et al. found a correlation between glutamine dependence and cancer invasiveness. Therefore, in their studies, high-invasive ovarian cancer cells are markedly glutamine dependent, whereas low-invasive OVCA cells are glutamine independent (30). Furthermore, GLS overexpression is associated with poor survival and it is associated with platinum resistance in ovarian cancer (31). GLS is not the only marker associated with glutamine metabolism; the glutamine transporter ASCT2 (SLC1A5) is actively investigated as a possible therapeutic target to block cancer cell growth and development (32). In order to support tumor expansion and the *de novo* production of amino acids, lipids and nucleic acid tumor cells present an increased request of glycine. The *de novo* serine synthesis pathway initiated by phosphoglycerate dehydrogenase has been considered as a hallmark of metabolic adaption in carcinogenesis (33). In any case, despite the strong evidence of the link between these metabolic dysregulations and cancer, none of these markers was associated with the survival of mice in our pilot study. However, we observed a negative correlation between the expression of MCT4 and that of FAS in PDX samples, suggesting that these two markers could underscore the prevailing glycolytic and oxidative metabolism, respectively.

To investigate the metabolic profile of cancer, very complex, and high-resolution techniques can be used, including liquid chromatography–mass spectrometry. Liquid chromatography allows the physical separation of the metabolites that are than analyzed with the high sensitivity of mass spectrometry. These tandem techniques can be used with biological samples like plasma or tumor cell lysates, allowing the tracking of disease progression (34). However, there are also some drawbacks of these techniques, including (1) the high cost, (2) the relatively low number of samples which can be simultaneously analyzed (thus incrementing the variability of the experiments), (3) the complex sample preparation and analysis, and (4) the requirement of freshly frozen tumor samples.

In contrast, IHC is an established technique available in all pathology units. Some *in situ* biomarkers, such as ER/PG receptors and HER2/neu in the case of breast cancer, have been used for decades for therapy stratification purposes (35), underlying the clinical value of IHC assessment of predictive biomarkers. The digital evolution of the analysis of IHC data offers an opportunity to overcome traditional limitations of this technique and enables the quantification of candidate metabolism-associated biomarkers to improve the prediction of disease aggressiveness and patient outcome (36). One limitation of our study is that we did not validate MCT4 as a prognostic biomarker in patients. Translational research demands a large cohort of patients to have enough power to draw solid conclusions. Along this line, we started the evaluation of the prognostic value of MCT4 expression in human ovarian cancer samples from the MITO2 clinical trial. MITO2 is a randomized, multicenter phase 3 trial conducted with 820 advanced ovarian cancer patients assigned with carboplatin/paclitaxel or carboplatin/PLD-pegylated liposomal doxorubicin as first-line treatment. Sixteen biomarkers were already studied in 229 patients in a tissue microarray (37), and additional biomarkers such as MCT4 can easily be analyzed. We foresee that the *in situ* metabolic panel presented in this pilot study will be useful to profile clinical samples in future studies.

## Data Availability Statement

The raw data supporting the conclusions of this article will be made available by the authors, without undue reservation, to any qualified researcher.

## Ethics Statement

The studies involving human participants were reviewed and approved by IOV Institutional Review Board and Ethics Committee (EM 23/2017), and were performed in accordance with the declaration of Helsinki. The patients/participants provided their written informed consent to participate in this study. This animal study was reviewed and approved by the Italian Ministry of Health (n. 217/2013-B).

## Author Contributions

IP contributed to the design of the study, performed the analysis, and wrote sections of the manuscript. MV collected the samples and performed the experiments. FM performed the experiments, analyzed the data, and wrote sections of the manuscript. GN, EZ, and TZ performed the experiments. PD performed the statistical analysis. GE analyzed the data and reviewed the manuscript. SI contributed conception and design of the study, wrote the first draft of the manuscript revising it critically for important intellectual content, and provide approval for publication of the content. All authors contributed to manuscript revision, read, and approved the submitted version. All authors contributed to the article and approved the submitted version.

## Conflict of Interest

The authors declare that the research was conducted in the absence of any commercial or financial relationships that could be construed as a potential conflict of interest.
