# Supplementary material for: In situ Metabolic Profiling of Ovarian Cancer Tumor Xenografts: A Digital Pathology Approach
Source: Front Oncol. 2020 Aug 19;10:1277. doi: 10.3389/fonc.2020.01277 (PMC7466758; doi:10.3389/fonc.2020.01277)
Supplement: Supplementary file 1 [file Table_1.docx]

**Supplementary Table 1.** Quantification of expression levels of metabolism-associated markers by digital pathology


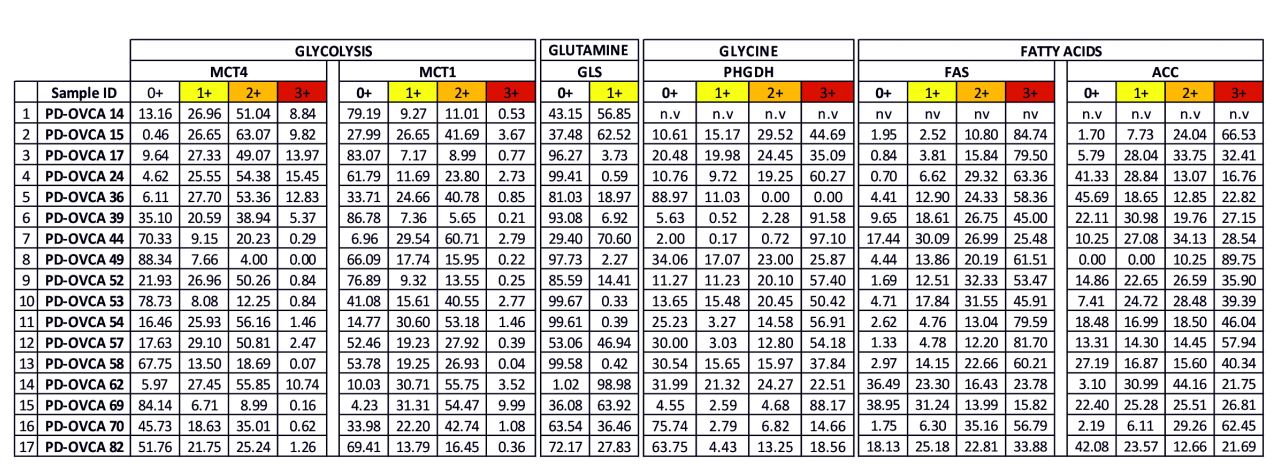


For all markers analyzed, the algorithm first identifies tumor cells and then quantifies expression levels according to a four-tier (MCT1, MCT4, PHGDH, FAS, ACC)

or a two-tier (GLS) classification system. The sum of the percentage of marker-positive cells for these tiers equals 100%.
