# Supplementary material for: In situ Metabolic Profiling of Ovarian Cancer Tumor Xenografts: A Digital Pathology Approach
Source: Front Oncol. 2020 Aug 19;10:1277. doi: 10.3389/fonc.2020.01277 (PMC7466758; doi:10.3389/fonc.2020.01277)
Supplement: Supplementary file 2 [file Table_2.DOCX]

**Supplementary Table 2.** Distribution of markers in the study population

| **Marker** | **N** | **Median (Q1;Q3)** |
| --- | --- | --- |
| **MCT4** | 17 | 51.1 (20.5;63.0) |
| **MCT1** | 17 | 28.3 (16.2;45.4) |
| **GLS1** | 17 | 19.0 (2.3;56.9) |
| **PHGDH** | 16 | 68.9 (48.4;78.0) |
| **FAS** | 16 | 17.2 (7.4;32.0) |
| **ACC** | 16 | 36.4 (31.0;49.0) |
| **pHH3** | 16 | 2.3 (1.7;3.0) |
| **MVD** | 15 | 88.5 (69.6;115.1) |
| **MVS** | 15 | 111.6 (95.7;170.6) |
